# Voxel Based Analysis of Surgical Revascularization for Moyamoya Disease: Pre- and Postoperative SPECT Studies

**DOI:** 10.1371/journal.pone.0148925

**Published:** 2016-02-11

**Authors:** Yasutaka Fushimi, Tomohisa Okada, Yasushi Takagi, Takeshi Funaki, Jun C. Takahashi, Susumu Miyamoto, Kaori Togashi

**Affiliations:** 1 Department of Diagnostic Imaging and Nuclear Medicine, Kyoto University Graduate School of Medicine, Kyoto, 606–8507, Japan; 2 Department of Neurosurgery, Kyoto University Graduate School of Medicine, Kyoto, 606–8507, Japan; Emory University School of Medicine, UNITED STATES

## Abstract

Moyamoya disease (MMD) is a chronic, progressive, cerebrovascular occlusive disease that causes abnormal enlargement of collateral pathways (moyamoya vessels) in the region of the basal ganglia and thalamus. Cerebral revascularization procedures remain the preferred treatment for patients with MMD, improving the compromised cerebral blood flow (CBF). However, voxel based analysis (VBA) of revascularization surgery for MMD based on data from pre- and postoperative data has not been established. The latest algorithm called as Diffeomorphic Anatomical Registration Through Exponentiated Lie Algebra (DARTEL) has been introduced for VBA as the function of statistical parametric mapping (SPM8), and improved registration has been achieved by SPM8 with DARTEL. In this study, VBA was conducted to evaluate pre- and postoperative single photon emission computed tomography (SPECT) images for MMD by SPM8 with DARTEL algorithm, and the results were compared with those from SPM8 without DARTEL (a conventional method). Thirty-two patients with MMD who underwent superficial temporal artery-middle cerebral artery (STA-MCA) bypass surgery as the first surgery were included and all patients underwent pre- and postoperative 3D T1-weighted imaging and SPECT. Pre- and postoperative SPECT images were registered to 3D T1-weighted images, then VBA was conducted. Postoperative SPECT showed more statistically increased CBF areas in the bypassed side cerebral hemisphere by using SPM8 with DARTEL (58,989 voxels; P<0.001), and increased ratio of CBF after operation was less than 15%. Meanwhile, postoperative SPECT showed less CBF increased areas by SPM8 without DARTEL. In conclusion, VBA was conducted for patients with MMD, and SPM8 with DARTEL revealed that postoperative SPECT showed statistically significant CBF increases over a relatively large area and with at most 15% increase ratio.

## Introduction

Moyamoya disease (MMD) is a chronic, progressive, cerebrovascular occlusive disease that causes abnormal enlargement of collateral pathways (moyamoya vessels) in the region of the basal ganglia and thalamus. Cerebral revascularization procedures remain the preferred treatment for patients with MMD [1], improving the compromised cerebral blood flow (CBF), reducing ischemic attacks, and resulting in sufficiently good long-term results [2]. However, in spite of established revascularization procedures, the method for evaluating the efficacy of revascularization surgery for MMD based on data from pre- and postoperative data has not been established.

Postoperative cerebral hyperperfusion syndrome has been considered to be less common in patients with MMD, because relatively low-flow revascularization can usually be attained surgically for MMD [3–5]. Revascularization surgery in patients with MMD carries a low risk, is effective at preventing future ischemic events, and improves quality of life [6, 7]. However, a recent study reported that cerebral hyperperfusion syndrome after superficial temporal artery (STA)-middle cerebral artery (MCA) bypass occurs more frequently in patients with MMD than in those with arteriosclerotic disease, with a diagnostic criteria of qualitative observation of focal intense CBF increase [8]. It is evident that favorable postoperative CBF increase as the effect of STA-MCA bypass should be differentiated from unwanted hyperperfusion syndrome.

Given these considerations, objective measurements are required to confirm the potential advantages of revascularization surgery, and many quantitative approach has been conducted [9]. On the contrary, voxel based analysis (VBA) is now widely performed, especially for magnetic resonance imaging (MRI) analysis. This technique enables fully automatic processing of images and is considered highly objective. Postoperative evaluation of MMD has been reported with dynamic susceptibility-weighted perfusion MRI [10], arterial spin labeling [11], computed tomography perfusion [12], and single-photon emission computed tomography (SPECT) [13], but those studies used region-of-interest analysis, and no VBA study has previously been conducted probably due to misregistration associated with postoperative brain shift. VBA for CBF changes for ischemia in the internal carotid artery area after bypass surgery was reported previously with relatively old algorithm [14]. With the advent of new algorithm such as the Diffeomorphic Anatomical Registration Through Exponentiated Lie Algebra (DARTEL), which has been introduced for VBA as the function of statistical parametric mapping (SPM8), improved registration accuracy has been brought to realization [15], VBA with the latest algorithm should be verified in pre- and postoperative images. This study was conducted to evaluate pre- and postoperative single photon emission computed tomography (SPECT) images for MMD by SPM8 with DARTEL algorithm, and the results were compared with those from SPM8 without DARTEL (a conventional method).

In this study, SPECT images of pre- and post-revascularization surgery for MMD were analyzed by VBA with DARTEL algorithm, and the results were compared with those from VBA without DARTEL (conventional method).

## Materials and Methods

### Patients

This retrospective study was approved by the institutional review board “Kyoto University Graduate School and Faculty of Medicine, Ethics Committee” and the need for written informed consent was waived. Patient information was anonymized and de-identified prior to analysis. Participants comprised 33 consecutive adult patients with MMD who underwent first STA-MCA bypass surgery at our institute between January 2010 and June 2013. The diagnosis of MMD was made in accordance with published guidelines for bilateral MMD [16, 17]. Patients with typical occlusive findings in the terminal portion of a unilateral internal carotid artery, which was diagnosed as probable MMD, were also included in this study (unilateral MMD), because asymmetry in the progression of stenosis is relatively common among patients with unilateral MMD [18]. Patients with autoimmune disease, Down syndrome or neurofibromatosis were excluded. Patients younger than 18 years old were also excluded, because age-related changes in cerebral blood flow (CBF) might occur, and STA-MCA bypass with encephalo-myosynangiosis was conducted for child patients instead of STA-MCA bypass surgery only [19]. Patients who had undergone previous bypass surgery or other operation were also excluded. Patients with intracranial hemorrhage and patients with cerebral infarction of greater than 1/3 of MCA territory, or major territorial infarction of anterior or posterior cerebral infarction were also excluded.

All patients in this study underwent preoperative imaging with both SPECT and MRI. All patients underwent STA-MCA bypass as the first surgery and the branches of the STA were anastomosed to the central branch of MCA by end-to-side anastomosis [20]. Postoperative SPECT and MRI were obtained on around postoperative day 5. All but 1 patient with postoperative focal cerebral infarction were included for comparisons between pre- and postoperative SPECT, and this patient was excluded from the study.

### Imaging Study

SPECT was acquired at resting state using a 2-head rotating gamma camera (Infinia; GE Medical Systems, Milwaukee, USA) with an extended low-energy general-purpose collimator. Patients were asked to keep the eyes closed during scanning. A bolus of N-isopropyl-p-[(123)I]-iodoamphetamine (^123^I-IMP) (167 MBq) with 10 ml of normal saline was administered intravenously at the beginning of image acquisition. Data were acquired in a 64 × 64 matrix through a 120° rotation at angle intervals of 4°. Total imaging time was 30 min. Spatial resolution at the center of view was 9.9 mm in full-width at half-maximum (FWHM) activity. Transverse reconstruction with Ramp and Butterworth filters (cutoff of 0.5 cycles/pixel and order 10), and attenuation correction using the method of Chang (0.07/cm) were applied. FWHM of the collimator was 10 mm.

Pre- and postoperative MR scans included 3-dimensional (3D) T1-weighted imaging, diffusion weighted imaging, 2D Fluid Attenuated Inversion Recovery (FLAIR) imaging and MR angiography using 3-T MR units (Magnetom Trio and Magnetom Skyra; Siemens, Erlangen, Germany), using 32-channel head coil. The parameter of 3D T1-weighted imaging was as follows: magnetization prepared rapid acquisition with gradient echo (MPRAGE) repetition time 1900 msec, echo time 2.58 msec, inversion time 900 msec, flip angle 9 degrees, field of view 230 × 230 mm, acquisition matrix 256 × 256, pixel size 0.90 × 0.90 mm, slice thickness 0.9 mm.

### Postimaging Analysis

**Fig 1:**
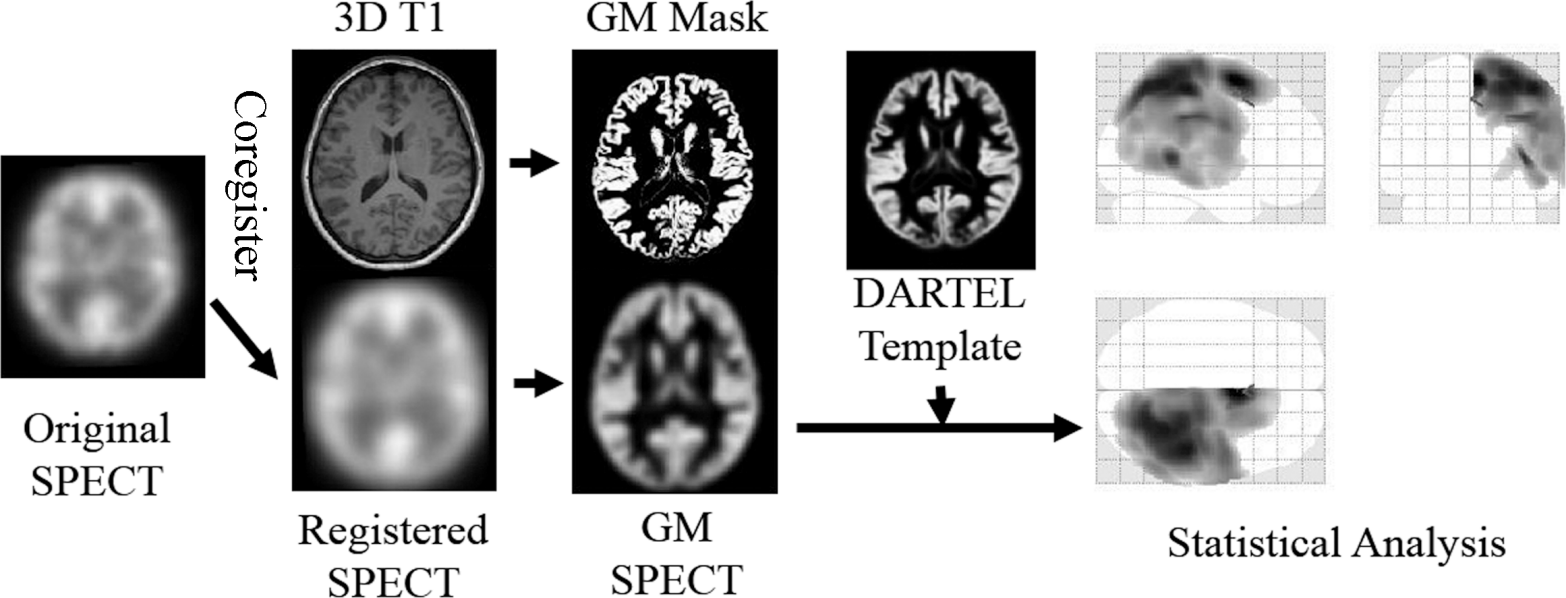
The flowchart of postimaging process of VBA. In the first place, horizontally flipping was conducted for the SPECT data with left side operation in order to deal with all the SPECT data as that with right STA-MCA bypass surgery. After global mean normalization was performed, pre- and postoperative IMP-SPECT images were co-registered to corresponding 3D T1-weighted images. The 3D T1 images were then segmented into gray matter (GM), white matter (WM) and cerebrospinal fluid (CSF) space using new segmentation tool and DARTEL import files were created for each patient. GM segment of coregistered SPECT (GM-SPECT) was created. Then, DARTEL template was generated with DARTEL import files. In the last place, non-linear warping of all GM images including GM-SPECT to MNI space was conducted with 2 methods as follows, with DARTEL by using flow field, or without DARTEL (a conventional method, which has been used in the previous literatures).

Because the patients underwent bypass surgery on either the left or right side, the operated side was made to appear as the right side by horizontally flipping left-side patient images. Thus all SPECT data could be dealt with as data from patients with right STA-MCA bypass surgery. The count of each voxel was normalized by the total count for the brain (proportional scaling of the global mean to 100). Pre- and postoperative IMP-SPECT images were co-registered to corresponding 3D T1-weighted images using statistical parametric mapping (SPM8) software (Wellcome Department of Imaging Neuroscience, University College London, UK) implemented on MATLAB 2013b (Mathworks, Natick, Massachusetts, USA). The 3D T1 images were then segmented into gray matter (GM), white matter (WM) and cerebrospinal fluid (CSF) space using new segmentation tool in SPM8, and DARTEL import files were created for each patient during this process [21]. A DARTEL template was generated from the entire image dataset using DARTEL import files. In the next step, the GM segment of coregistered SPECT (GM-SPECT) was created. Non-linear warping of all GM images to the Montreal Neurological Institute (MNI) space (http://www.mni.mcgill.ca/) was conducted with 2 methods as follows, (a) non-linear warping was conducted with DARTEL template and flow fields which store the deformation information, (b) non-linear warping was conducted without DARTEL (a conventional method, which has been used in the previous literatures), then the normalization parameters derived were applied to GM-SPECT, respectively. Spatially normalized GM-SPECT images were subsequently smoothed using an isotopic Gaussian kernel with a 12-mm full-width at half-maximum (Fig 1).

### Postimaging Analysis

**Fig 2:**
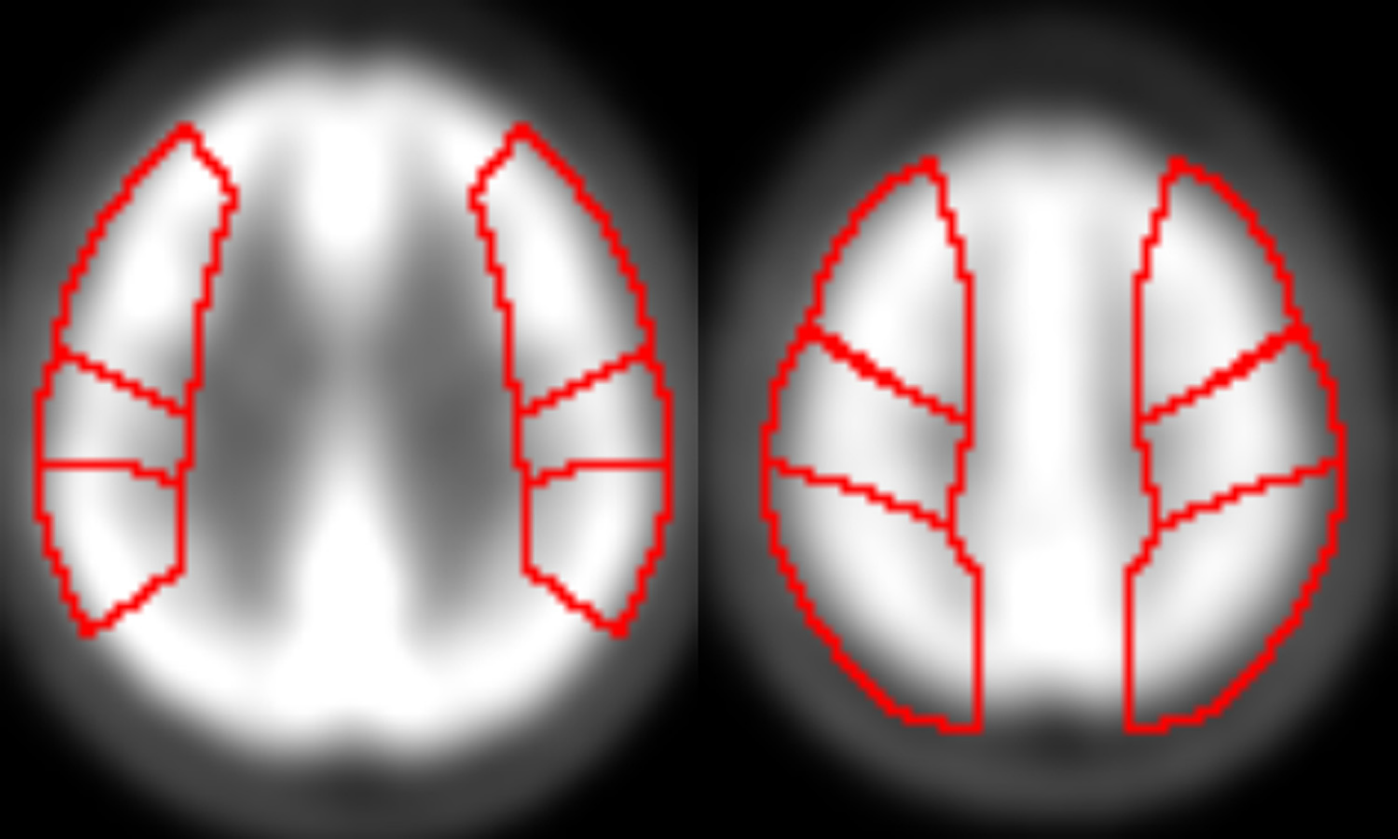
ROIs of bilateral precentral, central and parietal area. ROIs of bilateral precentral, central and parietal areas were created, and the same ROIs were applied for all cases.

ROI analysis was performed for each dataset of pre- and postoperative SPECT (GM-SPECT) in order to validate DARTEL method [22]. ROIs of bilateral precentral, central and parietal areas were created (Fig 2). Asymmetry index was created for the bypass side by using bilateral ROIs, then pre- and post-operative ROIs were compared.

### Statistical Analysis

Paired *t*-test was performed between pre- and postoperative GM-SPECT images voxel-by-voxel, and postoperative CBF increase were investigated. In SPM analysis, the P value threshold was 0.001 at the voxel level, and clusters were considered as significant when falling below a cluster-corrected p (FDR) = 0.05. Increased ratios (%) and decrease ratios (%) of postoperative CBF compared with preoperative CBF at areas of significant CBF increase were calculated and shown by using the xjView toolbox (http://www.alivelearn.net/xjview).

Paired *t*-test was also performed ROI values between pre- and postoperative GM-SPECT images. P value threshold < 0.05 was considered as significant.

## Results

In total, 32 hemispheres of 32 patients (9 males, 23 females; mean age, 38.2 years; range, 18–52 years) underwent STA-MCA anastomosis (bilateral MMD, n = 24; unilateral MMD, n = 8) (Table 1). The contralateral hemisphere was operated later in 12 of the 24 patients with bilateral MMD, but only the hemisphere of first surgery was analyzed in this study. Postoperative SPECT was conducted at a mean of 5.1 days (range, 2–11 days) after surgery.

**Table 1:** Profile of Patients Included in the Comparison of Pre- and Postoperative SPECT.

Initial Bypass Surgery	Bilateral MMD	Unilateral MMD
Right	16	6
Left	8	2

### SPM8 with DARTEL

**Fig 3:**
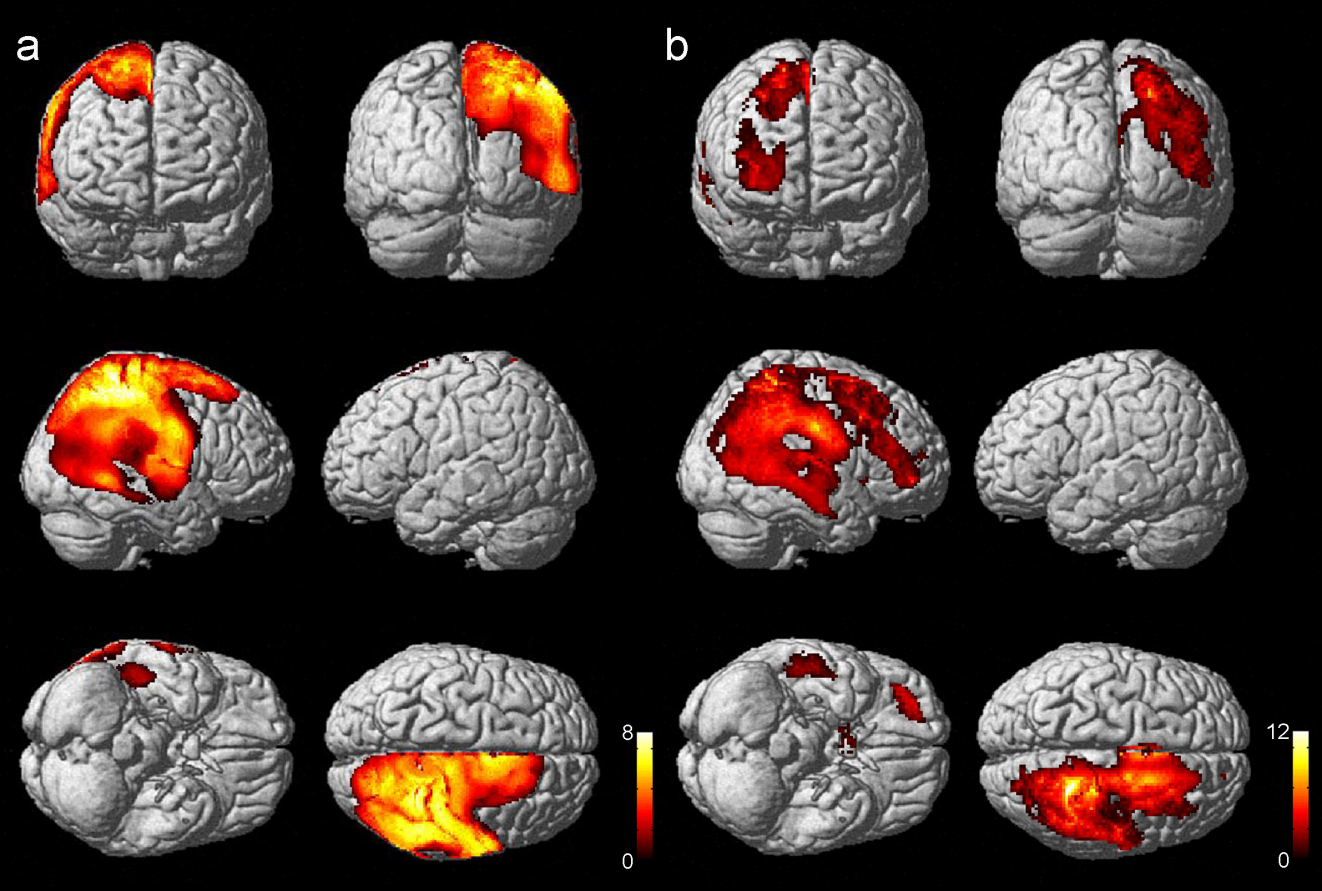
Postoperative CBF increased areas displayed in surface rendered images. Surface rendered images showed that significant postoperative CBF increased areas (58,989 voxels) in the right cerebral hemisphere or bypassed side by using SPM8 with DARTEL (a). These findings suggest that increased CBF due to STA-MCA anastomosis at the central branch of MCA appears not only at the site of anastomosis, but also in the wider area. Meanwhile, less significant areas (21,134 voxels) were shown by SPM8 without DARTEL (a conventional method) (b).

SPM results showed that significant postoperative CBF increases in the right cerebral hemisphere or bypassed side (58,989 voxels; Fig 3A), as the side of operation was depicted as the right side by flipping the images, as necessary. These findings suggest that increased CBF due to STA-MCA anastomosis at the central branch of MCA appears not only at the site of anastomosis, but also in the wider area.

**Fig 4:**
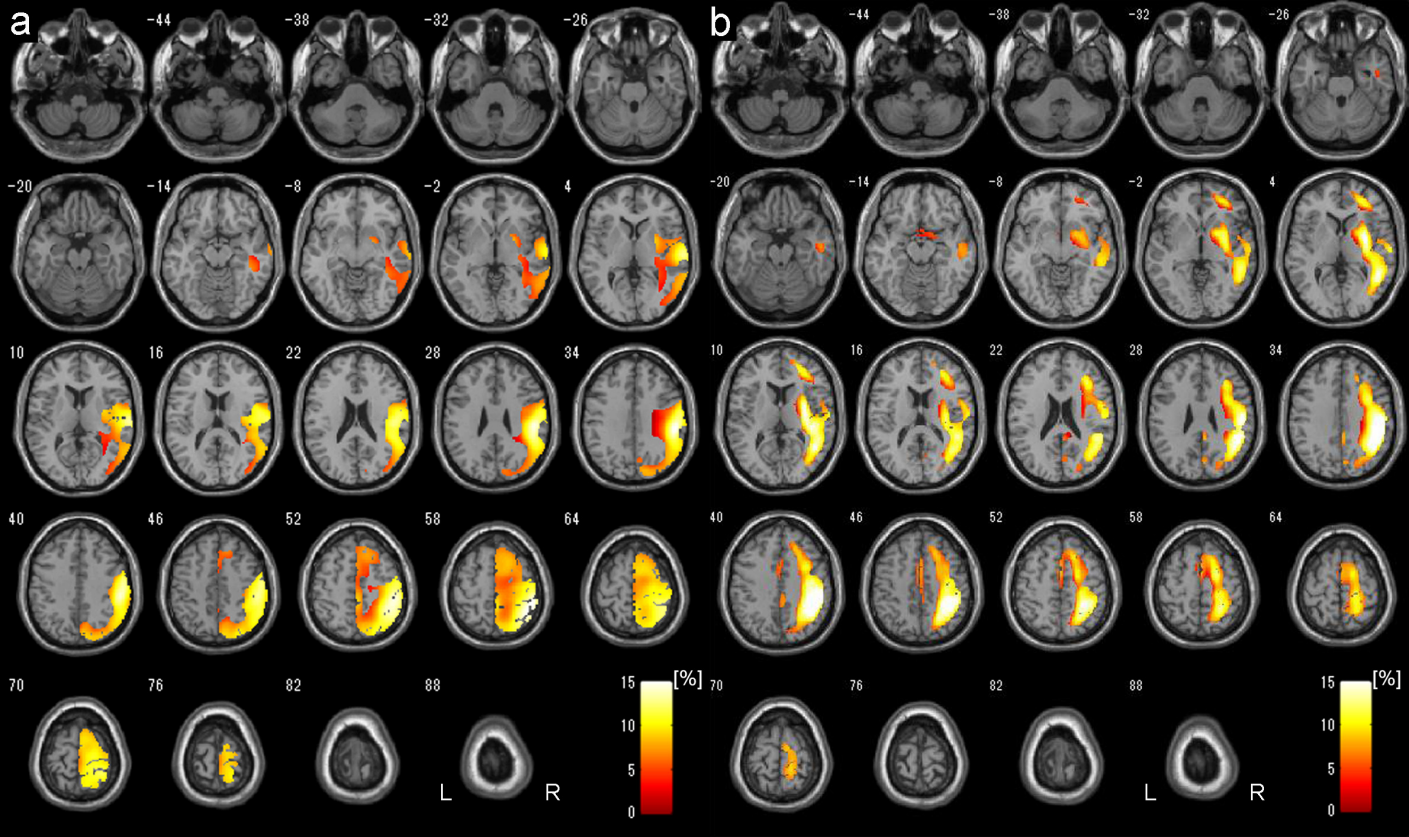
Postoperative CBF increased areas displayed in transverse sections. Increased ratio (%) at the area of significantly increased CBF on postoperative SPECT compared with preoperative SPECT is shown by using SPM8 with DARTEL (a) and SPM8 without DARTEL (a conventional method) (b). Images were reconstructed shown in 6mm thickness and the increased ratio was less than 15% in both methods. SPM8 with DARTEL revealed larger CBF increased areas (a), on the contrary, the conventional method showed relatively smaller CBF increased areas which were located in more medial regions compared with those with DARTEL.

Increased ratio (%) at the area of significantly increased CBF on postoperative SPECT compared with preoperative SPECT is shown in Fig 4A, and increased ratio was less than 15%.

**Fig 5:**
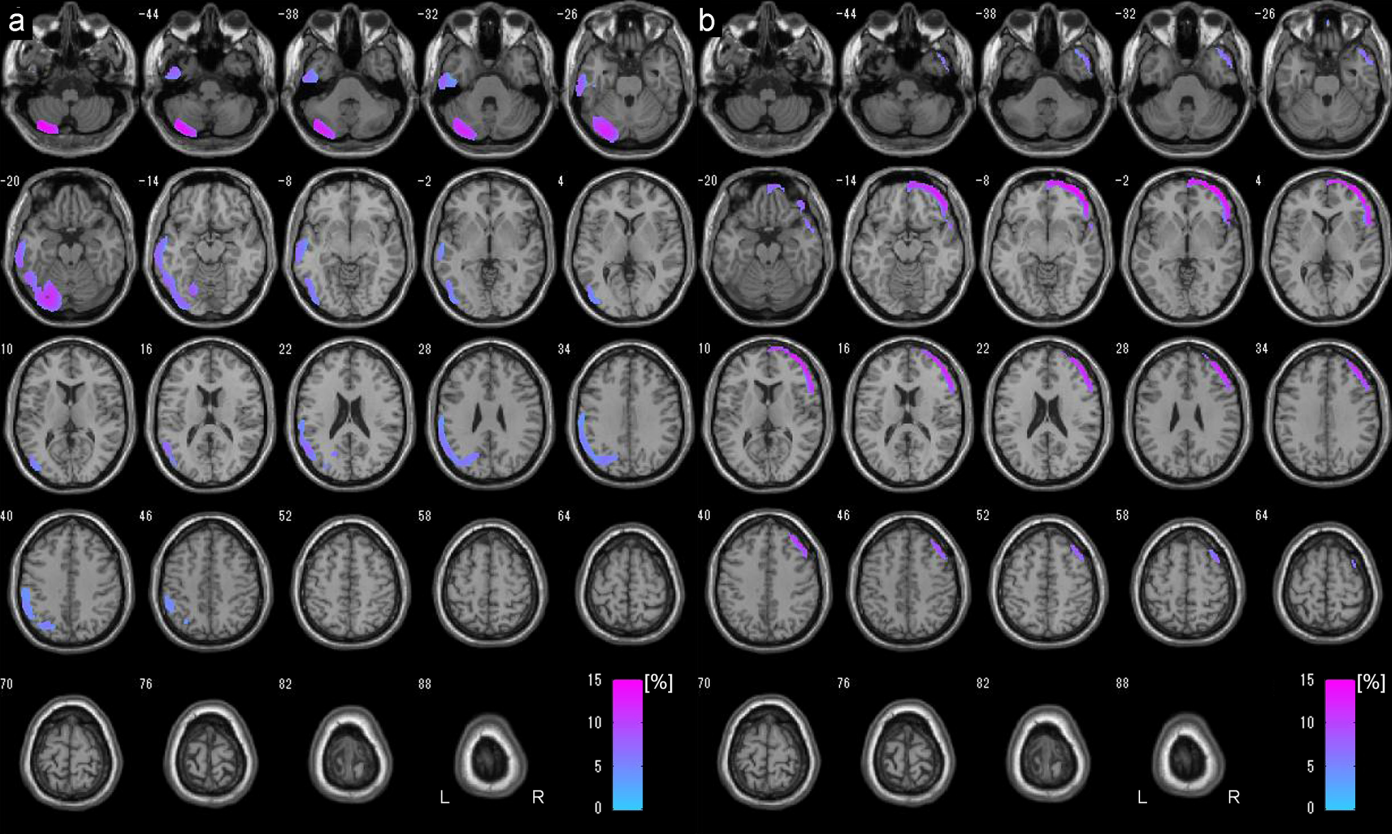
Postoperative CBF decreased areas displayed in transverse sections. Decreased ratio (%) at the area of significantly decreased CBF on postoperative SPECT compared with preoperative SPECT was shown in contralateral hemisphere to the STA-MCA bypass site by SPM8 with DARTEL (a). Since global mean normalization to the value of 100 was conducted for all SPECT data, ipsilateral CBF increase in wide area is likely to cause pseudo CBF decrease in the contralateral site. The conventional analysis showed significant decreased CBF areas on the surface of the ipsilateral hemisphere of bypass site (b). No decreased CBF areas were detected in the contralateral hemisphere, which suggests that decreased CBF areas in the operated hemisphere compensate CBF increase from the viewpoint of global mean normalization method.

Decreased ratio (%) at the area of significantly decreased CBF on postoperative SPECT compared with preoperative SPECT was shown in contralateral hemisphere to the STA-MCA bypass site (Fig 5A). Since global mean normalization to the value of 100 was conducted for all SPECT data, ipsilateral CBF increase in wide area is likely to cause pseudo CBF decrease in the contralateral side. Visual inspection was performed for all the individual SPECT data to make assurance, and no misregistration was present in the contralateral hemisphere.

### SPM8 without DARTEL (A Conventional Method)

SPM results showed that significant postoperative CBF increases in the right cerebral hemisphere or bypassed side (21,134 voxels; Fig 3B).

Increased ratio (%) at the area of significantly increased CBF on postoperative SPECT compared with preoperative SPECT is shown in Fig 4B. CBF increased areas were located in more medial regions, and relatively smaller than those with DARTEL. Increased ratio was less than 15%.

Decreased ratio (%) at the area of significantly decreased CBF on postoperative SPECT compared with preoperative SPECT was shown on the surface of the ipsilateral hemisphere of bypass site (Fig 5B). Apparent misregistration was not detected by visual inspection, however, slight medial shift of postoperative cerebral cortices derived from operation procedure compared with the preoperative MRI might induce mismatched comparison in MNI space between pre- and postoperative SPECT due to less accurate normalization to MNI space compared with DARTEL, because most counts of SPECT are derived from cerebral cortices. No decreased CBF areas were detected in the contralateral hemisphere, which suggests that decreased CBF areas in the operated hemisphere compensate CBF increase from the viewpoint of global mean normalization method.

The summary of statistically important CBF changes after bypass surgery is shown in Table 2.

**Table 2:** Summarys of Statistically Important CBF Changes after Bypass Surgery.

	CBF Increase		CBF Decrease	
	SPM8 with DARTEL	SPM8 without DARTEL (A Conventional Method)	SPM8 with DARTEL	SPM8 without DARTEL (A Conventional Method)
**Operated Side**	58989	21134	0	19230
**Contralateral Side**	0	0	14502	0

Note that the unit is voxels.

### ROI Analysis

**Fig 6:**
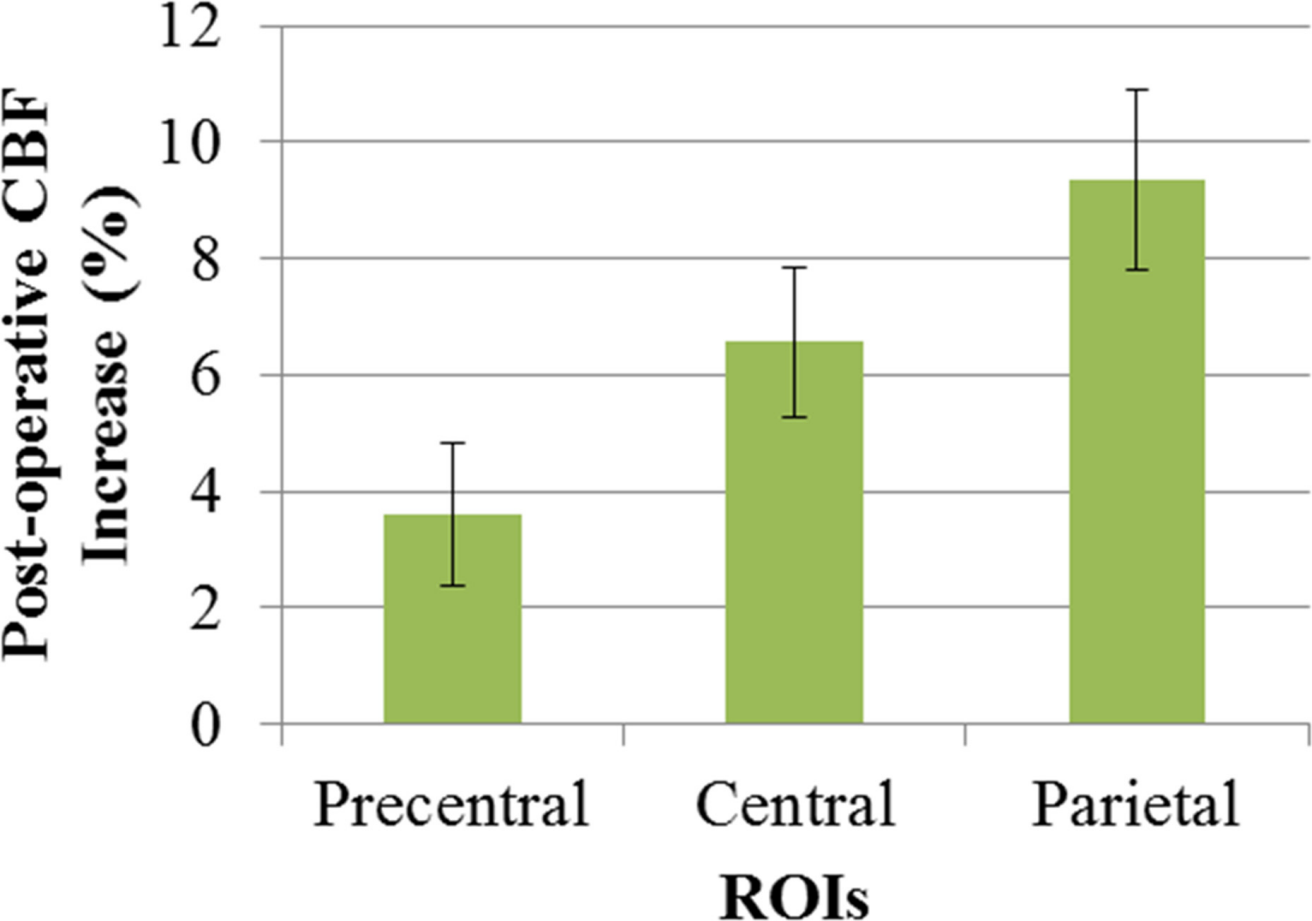
ROI analysis for post-operative CBF increase. Post operative CBF increase (%) at each ROI of bypass side are shown. The area near to the STA-MCA anastomosis showed higher increase ratio. Error bar represents standard errors.

ROI analysis also showed post-operative increase as shown as below and Fig 6. Results of ROI analysis were as follows: precentral 3.61 ± 1.23 (p = 0.007), central 6.57 ± 1.28 (p<0.001), and parietal 9.34 ± 1.55 (%) (p<0.001) (Fig 6).

## Discussion

Both VBA by using SPM8 with DARTEL and SPM8 without DARTEL showed ipsilateral CBF increase in the STA-MCA bypass side, and SPM8 with DARTEL showed larger CBF increased area (Figs 3 and 4). On the contrast, SPM8 without DARTEL showed significant ipsilateral CBF decrease at the surface of anastomosis site, which may suggest the focal misregistration probably due to postoperative changes in the images (Fig 5B). VBA for comparison between preoperative and postoperative SPECT successfully demonstrated CBF increase after STA-MCA bypass surgery, and DARTEL technique is considered to be robust against postoperative brain shift changes associated with STA-MCA bypass surgery.

Our study demonstrated a significant postoperative increase in a relatively large area of ipsilateral MCA territory in the operated hemisphere compared with preoperative SPECT, which demonstrated the therapeutic effect of direct STA-MCA bypass. Hyperperfusion syndrome was originally defined as a ≥100% increase from baseline without any time limit, to avoid missing late-onset hyperperfusion syndrome [23]. Qualitative IMP-SPECT has previously been used to demonstrate that radiological hyperperfusion occurs in 50% of patients with MMD after surgery using inner control of MCA/ipsilateral cerebellum ratio [13]. Our results with DARTEL technique suggest that moderate CBF increase at most 15% compared with preoperative SPECT occur due to improved blood supply from STA-MCA bypass in the postoperative state (Fig 4A).

Bypass surgery has been considered to play two important roles in MMD. One is to salvage misery perfusion, and the other is to reduce the fragile collateral vessels known as moyamoya vessels, because rupture of moyamoya vessels is thought to cause cerebral hemorrhage in MMD [6, 24]. The actual reductions in the risk of cerebral hemorrhage by STA-MCA bypass have been unclear [25], however, a recent study revealed that extracranial–intracranial bypass improved patient prognosis compared with non-surgical treatment, suggesting the preventive effect of direct bypass against rebleeding [26].

In this study, analysis was conducted using VBA with DARTEL technique, which provides improved registration accuracy compared with conventional VBA methods which had been used in the previous literatures [27]. Bypass surgery caused postoperative changes, but using anatomical MRI of patients with MMD and creating a template specific to patients with MMD by using DARTEL process based on the flow filed storing deformation information may allow precise registration and normalization for statistical comparison [15].

Several limitations to this study must be considered. First, the influence of bypass site on CBF has not been clarified. Slight brain shift might lead to comparisons between WM on preoperative SPECT and GM on postoperative SPECT; as a result, only GM on SPECT was analyzed in this study. Second, a correlation analysis with clinical data and individual patient analysis were not performed in this study. Since VBA has been less common in perioperative images, and more verification study might be necessary to establish VBA of longitudinal analysis for pre- and postoperative STA-MCA analysis. Third, cerebrovascular reserve was not analyzed in this study. Fourth, long-term follow-up exams were not included in this study.

In conclusion, VBA was conducted for patients with MMD, and SPM8 with DARTEL revealed that postoperative SPECT showed statistically significant CBF increases over a relatively large area and with at most 15% increase ratio.
